# Solid Organ Donation and Transplantation in the United Kingdom: Good Governance is Key to Success

**DOI:** 10.3389/ti.2023.11012

**Published:** 2023-05-25

**Authors:** Charlotte Johnston-Webber, Jasmine Mah, Apostolos Prionas, Simon Streit, George Wharton, John Forsythe, Elias Mossialos, Vassilios Papalois

**Affiliations:** ^1^ Department of Health Policy, London School of Economics and Political Science, London, United Kingdom; ^2^ Department of Medicine, Dalhousie University, Halifax, NS, Canada; ^3^ Department of Surgery, Imperial College, London, United Kingdom; ^4^ Department of General Surgery, Whipps Cross Hospital, Barts Health NHS Trust, London, United Kingdom; ^5^ Transplant Unit, Royal Infirmary of Edinburgh, Edinburgh, United Kingdom; ^6^ Institute of Global Health Innovation, Imperial College, London, United Kingdom; ^7^ Renal and Transplant Unit, Hammersmith Hospital, Imperial College Healthcare NHS Trust, London, United Kingdom

**Keywords:** organ donation, organ transplantation, transplantation policy, United Kingdom, UK

## Abstract

The United Kingdom (UK) supports a highly successful organ donation and transplantation program. While the UK originally had one of the lowest organ donation rates in Europe, sustained reforms have resulted in steady improvement. Of note, the UK nearly doubled its rate of deceased donations between 2008 and 2018. In this report, we present a case study of the UK organ donation and transplantation program as an example of a complete system with sound and inclusive governing structures that are strongly integrated with critical programs focused on training and research. This study was based on an initial targeted review of the literature led by a UK expert that included guidelines, national reports, and academic papers. Feedback solicited from other European experts was incorporated into our findings *via* an iterative process. Overall, the study highlights the stepwise evolution of the UK program that ultimately became successful largely due to ongoing collaborative efforts carried out at all levels. Centralized coordination of all aspects of the program remains a key driver of improved rates of organ donation and transplantation. The designation and empowerment of expert clinical leadership have helped to maintain focus and promote ongoing quality improvement.

## Graphical Abstract

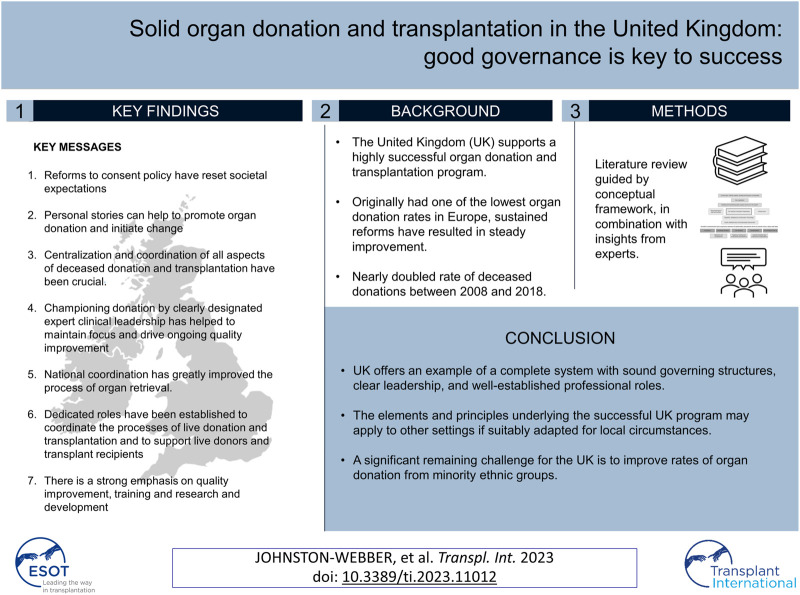

## Introduction

Although the United Kingdom (UK) spends considerably more on healthcare than some of the other European countries with successful organ donation and transplantation programs (e.g., Croatia, Portugal, and Spain (1–3)), it has only recently achieved success in this field. In the early 2000s, the rate of organ donation in the UK was among the lowest in all of Europe. However, largely as a result of a series of reforms that were instituted during the past two decades, organ donation rates in the UK have been increasing steadily. The deceased donation (DD) rate in the UK increased from 13 per million population (pmp) in 2008 (4) to 24.2 pmp in 2018/19 (5).

This paper outlines the key features of the UK solid organ donation and transplantation program. We will highlight the factors that have contributed to the near doubling of its DD rate, which has significantly reduced the number of patients awaiting transplants. We intend to (1) assess recent trends in organ donation and transplantation and (2), describe the critical features and developments that promoted positive change in the UK.

Table 1 provides an overview of key statistics regarding the UK healthcare system and population health status, including health spending *per capita*, key health factors of relevance to organ failure and the number of people on renal replacement therapy.

**TABLE 1:** Health system financing and population health in the UK: key statistics.

Health system	References
• Health spending *per capita*, EUR 2900; EU average, EUR 2884	(6)
• Health spending as a percentage of the gross domestic product, 9.6%; EU average, 9.8%	(6)
• Public spending as a percentage of the total health expenditure, 78.8%	(6)
• Out-of-pocket payments as a percentage of the total health expenditure, 16%; EU average, 15.8%	(6)
• Percentage of the population reporting an unmet need for medical care, 3%	(6)
**Health status**	
• Percentage of the population over 65 years of age, 18.1%; EU average, 19.4%	(6)
• Life expectancy, 81.3 years; EU average, 80.9 years	(6)
• Percentage of the population that smokes daily, 15.8%; OECD average, 16.5%	(7)
• Litres of alcohol consumed *per capita* per year, 9.7L; OECD average, 8.7L	(7)
• Percentage of the population that is overweight or obese (BMI >25), 64.2%; OECD average, 56.4%	(7)
• Individuals maintained on renal replacement therapy, incidence 122 pmp (England)	(8)
• Individuals maintained on renal replacement therapy, prevalence, 1,038 pmp (England)	(8)

EUR, euro; EU, European Union; OECD, Organisation for Economic Co-operation and Development; BMI, body mass index, pmp = per million population.

## Materials and Methods

Our study of the UK organ donation and transplantation program began with a narrative review of the literature. The searches for this review were conducted using combinations of the keywords “organ donation and transplantation” and “United Kingdom.” We retrieved several key documents and resources that were relevant to the national organ donation and transplantation system, including those that examined current legislation in the UK. We also performed thorough searches of the comprehensive websites maintained by the National Health Service Blood and Transplant (NHSBT), the UK’s National Transplant Organization (NTO), and the Human Tissue Authority (HTA), as well as related bodies and institutions, including the British Transplantation Society. References were hand-searched to obtain more detailed information. Keywords that were directly relevant to the major issues identified were used to perform a more focused review of the literature. This second review targeted databases that included Medline and Web of Science; internet search engines (e.g., Google Scholar) were also used to retrieve relevant peer-reviewed papers from the academic literature. The searches were not limited by year of publication, although papers written in languages other than English were excluded from further consideration. One researcher screened the titles and abstracts of these papers and identified those that were directly relevant to the objectives of this study. The reference lists of papers in this second set were also hand-searched for additional source material. This approach also facilitated the retrieval of relevant items from the grey literature, including international reports and reviews.

**FIGURE 1:**
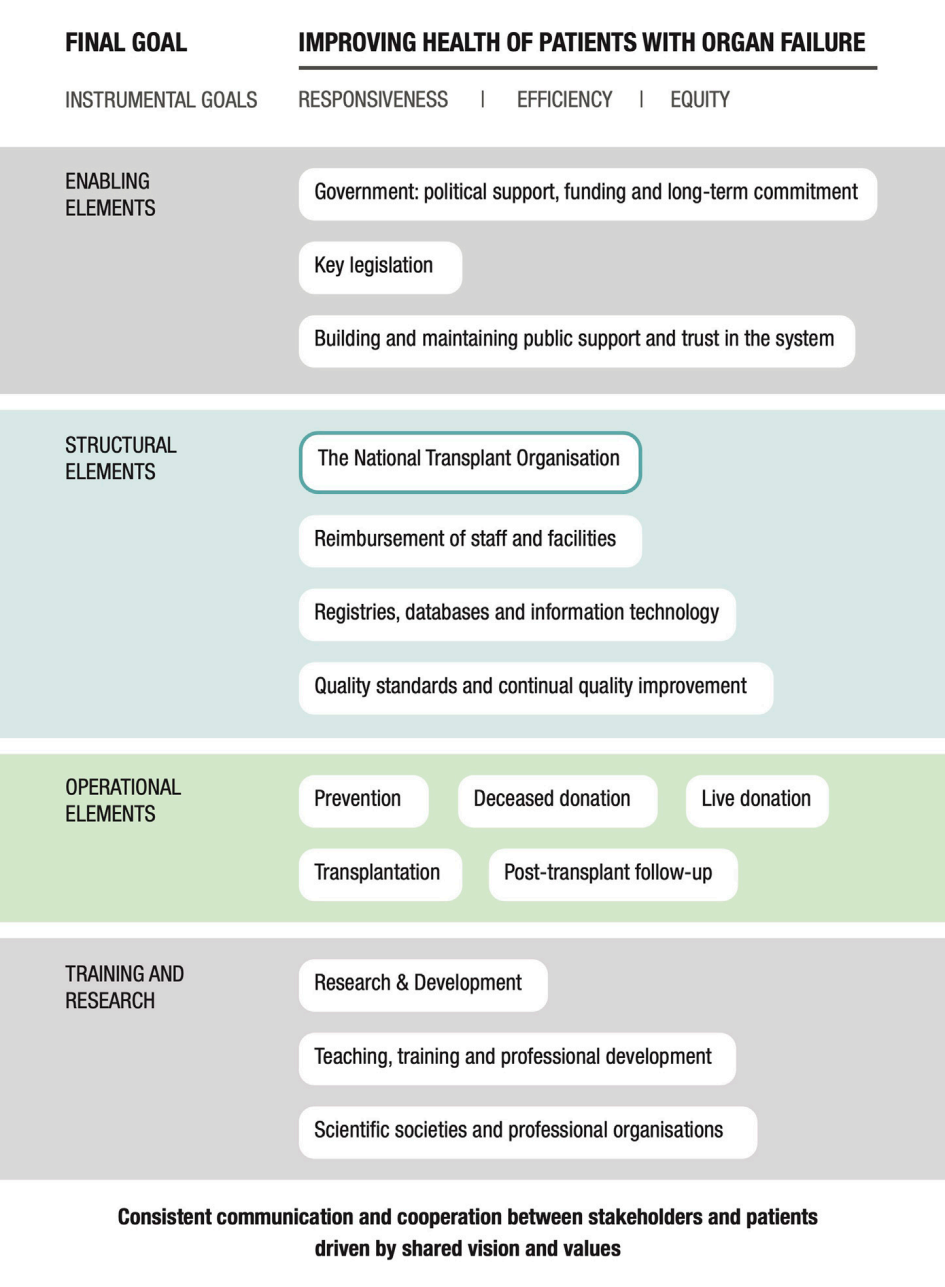
A conceptual framework used to understand and evaluate a national solid organ donation and transplantation program.

The next stage of the process was performed in consultation with a panel of international experts in organ donation and transplantation, including one expert from the UK. The case study was built according to the domains and elements included in the conceptual framework described in Johnston-Webber et al. (9) that featured the essential building blocks and goals of an organ donation and transplantation program (Figure 1). The expert panel provided feedback *via* an iterative process and reviewed consecutive drafts of the case study until all were satisfied that the findings presented were complete and accurate. The experts also suggested relevant resources that might supplement those identified by the aforementioned targeted review. The analysis focused on structures, processes and distinctive features of the system corresponding to domains of the framework, rather than performance in relation to health outcomes or health system goals.

The results presented below are consistent with the conceptual framework and represent a selection of the key features of the UK program that are relevant to our research objectives.

## Results

The UK organ donation and transplantation program has evolved in a step-wise fashion over a comparatively long period of time. Although many challenges remain, the developments in the UK highlight overall success with respect to this endeavour. These accomplishments are the result of many years of tireless campaigning, public engagement, legislative changes, system reviews, adaptation, and targeted reorganization. Several decades of sustained and coordinated efforts from all parties concerned have contributed to this achievement. Of note, there are currently considerable differences with respect to the organization, provision, and delivery of care by the NHS between the four UK jurisdictions (i.e., England, Scotland, Wales, and Northern Ireland). However, since 2005 a single organization, the NHSBT, has taken on the responsibility of coordinating and promoting organ donation and transplantation throughout the UK. The information presented in Table 2 provides a timeline and brief historical overview of the development of the UK program.

**TABLE 2:** Main developments in the UK program over the past 25 years.

1994	NHS organ donor registry is organized following a long public campaign to coordinate supply and demand
2000	UK Transplant is formed in 2000 and is tasked with increasing the number of organ donors
Early 2000s	The roles of donor liaison nurses, living donor coordinators, and regional transplant coordinators were established
2003	A transplant framework for England was published entitled “Saving Lives, Valuing Donors” (10)
2004–2005	Human Tissue Act legislation passed in 2004 led to the establishment of the Human Tissue Authority (HTA) in 2005. This authority was tasked with regulating all organizations involved in handling human tissue (Non-Departmental Public Body of the Department of Health and Social Care)
2005	UK Transplant merges with the National Blood Service in 2005 to become the National Health Service Blood and Transplant (NHSBT), a UK-wide Special Health Authority
2006	Human Tissue Act Scotland was enacted
2006–2008	The Organ Donation Taskforce (ODT) investigates and publishes a detailed report. A goal of increasing the deceased organ donation rate (then 13 pmp) by 50% by 2013 was set (4)
2010	In response to the ODT report, the National Organ Retrieval Service (NORS) is established as are the roles of Specialist Nurses in Organ Donation (SN-OD), Clinical Leads in Organ Donation (CL-OD), and Organ Donation Committees. The NORS, SN-OD and CL-OD posts are funded by the NHSBT
2011	National Institute for Health and Care Excellence (NICE) guidance entitled “Organ Donation for Transplantation—Improving donor identification and consent rates for deceased organ donation” was published (updated in 2016) (11)
2014/15	A review of ODT recommendations entitled “Taking Organ Transplantation to 2020” was published. A target of increasing the deceased donation rate from 19.1 pmp to 26 pmp by 2020 is set (12)
2015	Wales passes “soft opt-out” legislation (Human Transplantation [Wales] Act 2013) that was enacted in 2015 (13)
2020	England enacts “soft opt-out” legislation (Organ Donation [Deemed Consent] Act 2019) (14)
2021	Scotland enacts “soft opt-out: legislation (Human Tissue [Authorization] [Scotland] Act 2019) (15)
2023	Northern Ireland enacts “soft opt-out” legislation (Dáithí's Law) (16)

### Trends in Organ Donation and Transplantation in the UK

Prevention of organ failure is currently a priority in the UK; this may contribute to the comparatively low incidence and prevalence of patients maintained on renal replacement therapy in this country (17). The public health bodies of all four UK jurisdictions run regular public information and education campaigns aimed at promoting healthy lifestyles and providing warnings about the harmful effects of smoking and excessive alcohol use. Primary and secondary school curricula cover topics that include healthy eating, exercise, and sexual health (18). All UK adults between 40 and 74 years of age are invited to visit their general practitioners for a health check every 5 years. Health checks routinely include discussions focused on lifestyle and general health as well as specific screening for diabetes, hypertension, hypercholesterolemia, and obesity (19). The UK has a robust primary care network and a voluntary annual reward and incentive program known as the Quality and Outcomes Framework (QOF). This program incentivizes primary care physicians to perform regular screenings and monitor the development of risk factors that might lead to organ failure, including hypertension, diabetes, and chronic kidney disease (20).

**FIGURE 2:**
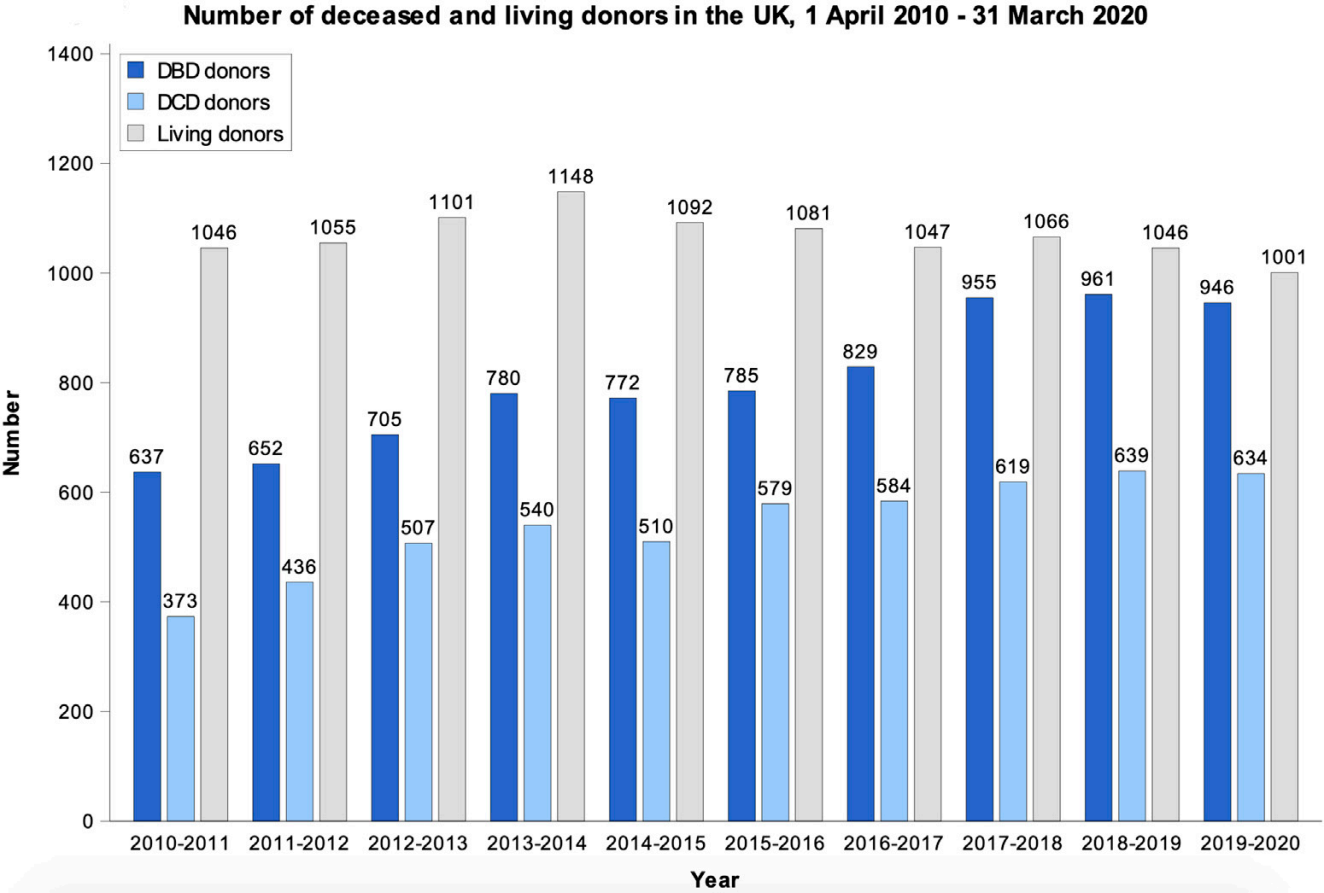
Data from the NHSBT Organ Donation and Transplantation Activity Report 2019/20 showing the absolute numbers of DBD, DCD and Living donors in the UK from April 2010 to March 2020 (21).

There has been considerable improvement in organ donation and transplantation in the UK over the past two decades. Possibly the most important factor in achieving this improvement was the work of the Organ Donation Taskforce, whose report detailing 14 recommendations was published in 2008(4). In this study we highlight some of these recommendations. However, it is important to point out that the 14 recommendations were intended to work as a group, and all 14 have been crucial to the improvements which were achieved in the UK program. Although the UK did not quite reach the target set by the “Taking Organ Transplantation to 2020” strategy of 26 DDs pmp by 2020 (12), the DD rate increased from 13 pmp in 2008 (4) to 24.2 pmp in 2018/2019 (5), albeit with a slight drop to 23.8 pmp in 2019/2020 (21). The UK is also a leader in the efforts to promote donation after circulatory death (DCD); these efforts are supported by a consensus statement from the British Transplantation Society and the Intensive Care Society (22), a Code of Practice for the Diagnosis and Confirmation of Death from the Academy of the Medical Royal Colleges (23), and legal guidance issued by the Department of Health in England and Health and Social Care Directorates in Scotland (24, 25). These documents have helped to reassure ICU clinicians that it is entirely appropriate to include consideration of organ donation as part of every end-of-life care pathway. The practice of DCD has grown steadily over the past decade, and now is the source of ∼40% of all DDs, a percentage that is higher than that reported by many comparable countries. Most of these donations involved controlled DCD in intensive care units that were initiated following decisions to withdraw life-sustaining treatments (26). Increasing utilisation of *ex-situ* normo- and hypothermic machine perfusion and *in-situ* normothermic regional perfusion techniques may have also helped to boost the rates of viable organ donations particularly from DCD and extended criteria donors (27, 28). An additional factor in ensuring sustained improvement in the rates of deceased donation has been the implementation of comprehensive reimbursement schedules for all deceased donor care expenses. This ensures that there is no financial burden on participating hospitals which could present a disincentive to undertake these procedures. Figure 2 shows the absolute numbers of DBD, DCD and living donors across the UK from April 2010 to March 2020.

**FIGURE 3:**
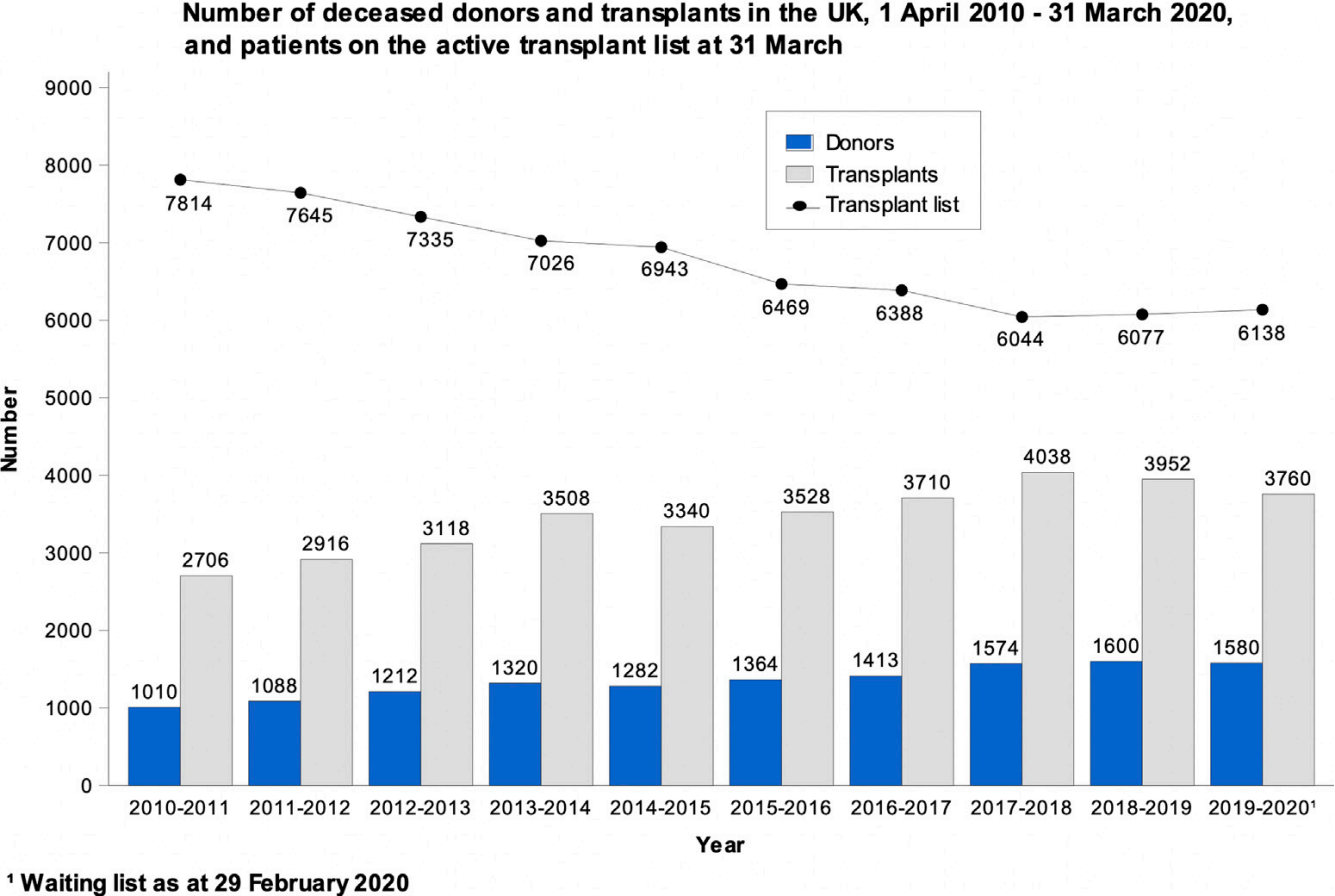
Data from the NHSBT Organ Donation and Transplantation Activity Report 2019/20 showing the absolute number of deceased donors, number of people on the waiting list and transplants performed in the UK from April 2010 to March 2020 (21).

Transplants have increased considerably over the years. In 2010/2011, 2706 organ transplants were performed in the UK. The number of transplants increased to a high of 4038 in 2016/2017, followed by a slight drop to 3760 in 2019/2020. The number of people on the waiting list dropped from 7814 in 2010/2011 to a low of 6044 in 2017/2018; this was followed by a small increase to 6138 in 2019/2020 (Figure 3). The onset of the Coronavirus disease-2019 (COVID-19) pandemic most likely had some impact on the rates reported for 2019/2020 as the NHSBT report covers through the end of March of this and each calendar year. Data for the remainder of 2020 onwards have not been included for the purposes of this case study as it has been greatly distorted due to the impact of the COVID-19 pandemic on the normal functioning of the organ donation and transplantation program.

UK-wide family consent rates are also improving slowly and have grown from 62.8% in 2016/2017 to 67.7% in 2019/2020 (both donation after brain death [DBD] and DCD). However, there remain considerable differences in consent rates across the UK. The consent rate in Wales reached 69%, while that in Northern Ireland remained at 62% during 2019/2020 (21). We note that Wales was the first of the four UK jurisdictions to enact “soft opt-out” legislation. Since 2015, Wales has been operating under the Human Transplantation (Wales) Act 2013 (13) which served to amend the 2004 Human Tissue Act and states that one assumes that an individual consents to donation unless otherwise stated, although family members can still register objections. England followed with similar legislation in 2019 (enacted in April 2020) (14). Similarly, in July 2019 the Scottish Parliament passed the Human Tissue (Authorization) (Scotland) Act 2019 (15) which also introduced a “soft opt-out” system; this was enacted in March 2021. Northern Ireland was last to make this change, with “soft opt-out” legislation enacted in spring 2023 (16).

The UK’s active living donor (LD) program contributes substantially to the overall transplant rate. In 2019/2020, the UK performed 14.8 LD transplants pmp. Recent data from the NHSBT reveals that 39% of donors are living at the time of donation; these donations account for 21% of all transplant activity. There is also a successful and growing UK-wide kidney exchange scheme (29). This program, which was established in 2007, celebrated its 1000th kidney transplant in 2019 (30).

### Key Elements of UK Policy Leading to Programmatic Reform

The following sections present the critical elements contributing to the reform of the UK organ donation and transplantation program. Table 3 provides a summary of these elements aligned with the elements of the conceptual framework described in Johnston-Webber et al. (9).

**TABLE 3:** Key elements of UK policy that have driven reform. These elements have been aligned with elements of the conceptual framework described in Johnston-Webber et al. (2021) (9).

Framework domain	Key features	Details
Enabling Elements: Government: Key Legislation	Reforms to consent policy have reset societal expectations	• All the constituent countries of the UK have now moved to a “soft opt-out” consent policy
Enabling Elements: Building and Maintaining Public Support and Trust in the System	Personal stories introduced in public information and education campaigns can help to promote organ donation and initiate change	• Moving stories of organ donors and transplant recipients have been effective in the UK setting
Structural Elements: National Transplant Organization (NTO)	The NTO provides centralization and coordination of all aspects of deceased donation and transplantation	NHSBT oversees and coordinates all aspects of deceased organ donation and transplantation *via*
		• Twelve regional organ donation teams, each serving several specific NHS Trusts and/or Boards
		• Eight solid organ advisory groups that include clinicians, scientists, and operational managers
	Championing donation by clearly designated expert clinical leadership has helped to maintain focus and drive ongoing quality improvement	• Specialist Nurses in Organ Donation (SN-ODs) and Clinical Leads in Organ Donation (CLODs) work closely with local Organ Donation Committees
	National coordination of the retrieval process	• The National Organ Retrieval Service (NORS) coordinates the retrieval process and thus prevents unnecessary delays
		• NORS currently includes ten abdominal and six cardiothoracic surgical teams
Structural Elements: Quality Standards and Continual Quality Improvement	A strong emphasis on quality improvement	• Comprehensive data are collected to cover all activities from donor identification to transplant follow-up
		• All units provide regular activity reports. The NHSBT publishes annual national reports and benchmarking data
		• The HTA audits and licenses transplant establishments
		• The HTA provides a platform for reporting serious incidents, as well as issuing alerts and warnings
Operational Elements: Living Donation and Transplantation	Dedicated roles established to coordinate the processes of live donation and transplantation and to support live donors and transplant recipients	• Transplant Recipient Coordinators (TRCs) support potential recipients
		• Living Donor Coordinators support living donors and organ recipients and promote living donation in their regions
Training and Research: Teaching, Training, and Professional Development	A strong emphasis on training for all staff involved in organ donation and transplantation	• All staff are expected to participate in tailored basic training and regular updates including instruction in specific communication skills
		• All staff members are expected to maintain a continuing professional development (CPD) portfolio and undergo regular review and appraisal
		• Advanced training opportunities are available
		• Junior staff members receive appropriate supervision
Training and Research: Research and Development (R&D)	A strong emphasis on research and development (R&D)	• The NHSBT maintains a designated research Advisory Group known as the Research Innovation and Novel Technologies Advisory Group (RINTAG)
		• RINTAG produces annual reports on relevant research activity

### Enabling Elements

#### Government: Key Legislation

##### Reforms to Consent Policy Have Reset Societal Expectations

“Opt-out” legislation has now been adopted across the UK (13–16). This has changed how conversations about DD are initiated and framed. This effectively facilitates the job of SN-ODs and improves the likelihood of obtaining consent. Wales led the way in enacting this legislation, which appears to have had a net positive effect. Rates of family consent and authorization as well as DD rates in Wales have increased since this legislation was enacted (31). Due to the impact of the COVID-19 pandemic, this paper only presents data up to early 2020, and therefore it is not possible to reflect on whether the changes to the legislation in the other devolved nations have had a similar effect. Each legislative change was preceded by a comprehensive consultation process, including surveys of public perceptions and opinions (32–35). This process was designed to raise the profile of organ donation and transplantation as well as to ensure that there were no unintended consequences of the legislation.

#### Building and Maintaining Public Support and Trust in the System

##### Personal Stories Can Help to Promote Organ Donation and Initiate Change

The recent change to the consent legislation in England has become known as “Max and Keira’s Law” after the young girl who was fatally injured in a car accident and the boy who received her donated heart. With the consent of their families, the *Mirror* newspaper ran a prominent and successful campaign which ultimately led to the change in the law of England to an “opt-out” policy (36). Personal accounts that help the public to identify with the poignant mixture of tragedy and joy in situations such as these appeared to have a significant impact in the UK.

### Structural Elements

#### The National Transplant Organization (NTO)

##### The Impact of Centralization and Coordination of all Aspects of Deceased Donation and Transplantation

Although the NHSBT was established in 2005, many managerial functions remained at the local level. Individual NHS Trusts and Boards were tasked with the responsibility of recruiting coordinators and allied staff members. One key message of the 2008 ODT was that an integrated UK-wide service was essential for effective oversight of all aspects of organ donation and transplantation and that this was a pre-requisite toward efforts to increase the DD rate (4). The NHSBT has now taken on this role and currently has overall responsibility for every step of deceased organ donation and transplantation. The NHSBT works closely with the HTA and the transplant centres, and, in the case of living donation, the HTA authorises all living donations, and ensures that the required standards have been met. The NHSBT maintains the organ donation registry and national transplant database, employs and provides professional development for specialist nurses, clinical leads, and organ retrieval teams, and establishes local Organ Donation Committees. There is a three-tier system that permits the NHSBT to oversee 12 regional organ donation teams, which in turn provide services to their designated populations and cover several NHS Trusts and Boards. Eight solid organ advisory groups (37) were also established; these groups are critical contributors to the success of the UK program, as they provide a forum at which clinicians, scientists, commissioners, and representatives of the government departments and directorates of health can meet regularly to discuss the current situation and future plans. The advisory groups include lay and patient representation. One key responsibility is to develop the selection, registration and allocation policies for their area of expertise, and these are approved by the Transplant Policy Review Committee acting on behalf of NHSBT. There are specific and detailed selection and allocation policies for each organ (38) and these are based on two key principles; ensuring equity of access to listing for transplantation and achieving the best possible outcomes. The lay and patient representatives of the solid organ advisory groups play an important role in the development of the allocation policies and their participation is vital to how the policies are perceived as fair and helps to protect them from unreasonable external challenges.

##### Championing Donation by Clearly Designated Expert Clinical Leadership has Helped to Maintain Focus and Drive Ongoing Quality Improvement

Consistent with the recommendations of the ODT report (4), the UK has developed clear clinical roles and leadership hierarchies in hospitals involved in deceased organ donation and retrieval. Specialist Nurses in Organ Donation (SN-ODs) are recruited, employed, and remunerated directly by the NHSBT and provide a round-the-clock service to all hospitals that participate in deceased organ donation. The SN-ODs receive rigorous training in communication skills and family support and thus play key roles in identifying all potential donors and facilitating organ donation and retrieval. The SN-ODs also provide teaching and training to colleagues and are responsible for ensuring that audits, policies, and resources are fully up to date. Additionally, they are responsible for gathering data for the annual Potential Donor Audit Report and inputting this to the NHSBT databases (39). This report gathers data on potential donations and the reasons for which potential donors do not become actual donors. It has been a crucial tool in increasing the number of donations in the UK and validating the presence of SN-ODs in the ICUs (and in other critical care settings).

SN-ODs are supported by Clinical Leads in Organ Donation (CLODs). The CLODs are usually senior physicians that have specialized training in intensive care or emergency medicine and are under a mandate to promote and champion organ donation in their localities. By 2017, 240 CLODs were operating in the UK and were responsible for covering all acute Trusts and Boards (40). CLODs are expected to commit a specific amount of time to the role for which they are reimbursed by the NHSBT. A network of regional CLODs has been established to promote professional support and development on a routine basis, as well as regularly-scheduled regional and national meetings at which all senior staff members are encouraged to attend.

Following the 2008 ODT report, Local Organ Donation Committees were established in every acute Trust or Board. These committees promote and endorse DD in their locality with a primary focus on performance, policy, education, and public promotion. The committee chair is a voluntary role, and this role is fulfilled by individuals from many different backgrounds. The chair works in close collaboration with the other committee members, in particular, local CLODs and SN-ODs (41).

In addition to the local Organ Donation Committees, there is a National Organ Donation Committee, this provides advice and guidance to the NHSBT and serves as the national representative body for the 12 UK regional organ donation teams. The National Organ Donation Committee meets three times a year and members include regional clinical leads and managers as well as senior staff from the ODT directorate of the NHSBT (42).

Organ donation and transplantation have been championed by establishing the roles of the SN-OD, the CLOD, and the Organ Donation Committee in every hospital Trust. Dedicated clinical leadership has removed barriers to organ donation and transplantation, promoted awareness, and fostered public acceptability. Regularly-scheduled meetings and opportunities for professional development promote a culture of collaboration and facilitate cohesion between the many different professionals involved in this process. Importantly, the SN-OD and CLOD posts are centrally funded by the NHSBT so that they are unlikely to be subsumed into other areas of need in the acute care environment.

##### National Coordination of the Retrieval Process

Before the National Organ Retrieval Service (NORS) was established in 2010, several surgical teams would often be involved simultaneously with a single donor. This frequently led to delays and problems with coordination. Similarly, other clinical commitments would divert focus from the donor and potentially compromise organ viability. The establishment of the centralized NORS, with on-call teams available round-the-clock, has greatly improved the process of organ retrieval. The NORS is commissioned and funded by the NHSBT, thus eliminating any controversy regarding reimbursement for organ retrieval (43).

#### Operational Elements

##### Dedicated Roles Established to Coordinate the Processes of Live Donation and Transplantation and to Support Live Donors and Transplant Recipients

###### Live Donation

Living Donor Coordinators are employed by all NHS Trusts involved in live donation to promote and facilitate living donation as well as guide living donors and their recipients through the transplant process.

###### Transplantation

On the transplant side of the process, Transplant Recipient Coordinators (TRCs) support potential recipients. There are currently 250 TRCS employed by NHS Trusts and based at 27 transplant units throughout the UK. These individuals play a critical role in the assessment process and coordinating the transplant waiting list. TRCs also educate patients and their families and coordinate the transplant procedure(44).

#### Quality Standards and Continual Quality Improvement

##### A Strong Emphasis on Quality Improvement

All Trusts, Boards, and their constituent hospitals provide detailed reports and updates on their activities on a regularly-scheduled basis and these are collated into an annual activity report by the NHSBT (21). SN-ODs are expected to conduct regular internal audits and collect data on missed potential donors and refusal rates. The NHSBT publishes benchmarking reports that compare the performances of individual Trusts and Boards to one another and highlight donor referral rates and the involvement of SN-ODs in donation discussions. The outcomes and pathways of all potential donor organs are recorded and charted from point of donor eligibility through successful transplantation. Reasons for organ non-retrieval or non-use are also documented, and outcome data are collected for several years post-transplant. Reports of selected serious incidents and examples of excellence are shared to promote learning and inform changes in practice.

Established in 2005, the HTA regulates organ donation and transplantation throughout the UK under The Quality and Safety of Organs Intended for Transplantation Regulations 2012. These regulations transfer the European Union Directive 2010/53/EU on the Standards of Quality and Safety of Human Organs Intended for Transplantation into UK law. Both procurement and transplantation activities are covered by this legislation. In the case of living donation, authorisation must be obtained *via* the HTA which provides independent checks to protect living donors and ensure that their desire to donate is free from any form of coercion. The HTA inspects, audits, and licenses all facilities that participate in organ donation and transplantation activities. The HTA also provides a platform for reporting serious incidents and/or adverse reactions linked to donation or transplantation activities and issues relevant alerts and warnings.

### Training and Research

#### Teaching, Training, and Professional Development

##### A Strong Emphasis on Training

The ODT report also recommended the need to strengthen training and education and provide ongoing educational support (4); providing and monitoring training is a core responsibility of the NHSBT. The NHSBT, the British Transplantation Society, and several Royal Colleges offer regularly-scheduled seminars, conferences, and training opportunities focused on organ donation and transplantation.

All healthcare professionals involved in organ donation and transplantation are expected to have an up-to-date CPD portfolio and personal development plan and are subject to routine appraisal and revalidation. These individuals are expected to complete basic training appropriate to their roles, and participate in regular educational updates and ongoing professional development. Advanced communication skills, including bereavement and family support, are understood to be crucial components of staff training; the NHSBT provides simulation training courses for all staff members involved in DD (45). SN-ODs are registered nurses, usually with a background in intensive care or trauma and emergency who have undergone 6 months of intensive clinical training for their role on the transplant team. CLODs are senior physicians with training in intensive care or trauma and emergency. These individuals are appointed for 3 years and are expected to participate in a two-day induction within the first year of their appointment.

The London Deanery currently offers specialty rotations in transplant surgery to post-graduate surgical trainees. As part of this program, trainees complete at least 4 years of general surgical training and 2 years of transplant surgical training including organ retrieval. On completion, fellowships are available that provide experience in more complex procedures, including live donor nephrectomies, pancreas transplantation, and liver transplantation (46).

#### Research and Development

##### A Strong Emphasis on Research and Development (R&D)

Research and development (R&D) are fully integrated into the NHSBT. These activities are perceived as crucial support for long-term improvements in patient outcomes. A national strategic plan supports an innovative translational program of research activities (47); a dedicated research office and R&D committee publish yearly reports and provide strategic oversight of these activities. The NHSBT Clinical Trials Unit supports researchers through the processes involved in conducting research, from the inception of ideas to dissemination of results (48). All research projects which use donation resources must be approved by The Research Innovation and Novel Technologies Advisory Group (RINTAG) of the NHSBT (49). RINTAG also promotes and supports research activities and ensures overall good governance of all research processes. Before commencement, all projects must be approved both by RINTAG and by the NHS Health Research Authority Research Ethics Committees (REC).

### Remaining Challenges

Despite these improvements, there remains a substantial unmet need in the UK. At the beginning of 2020, more than 6,000 people remained on waiting lists for all organ transplants, and three hundred and seventy-seven individuals died in 2019/2020 while awaiting transplants (21). The average waiting time for a kidney transplant in 2019/2020 was 633 days, although this number had fallen from 706 days reported in the previous year.

Rates of donation and family consent from minority ethnic groups are improving but remain problematic. There is a greater likelihood that a matching organ will be found from a donor with the same ethnic background as the recipient. At the same time, ethnic minorities in the UK are more likely to develop hypertension, diabetes, and some forms of hepatitis, thus increasing their susceptibility to organ failure. In 2019/2020 only 7% of the deceased organ donors were members of ethnic minority groups. By contrast, 25% of the transplants were performed on these recipients. On average, Black patients will wait almost a year longer to receive a kidney transplant than white patients (50).

Another challenge is the comparatively few critical care beds available in the UK together with the high occupancy of critical care units. Despite recent increases, the number of critical care beds per 100,000 population in the UK remains consistently low when compared to similar resources available in other equivalent economies (51).

The UK has recently experienced a profound setback with the loss of the UK Donation Ethics Committee (UKDEC), which was a group established in 2009 in response to the aforementioned ODT report. The UKDEC helped to address difficult ethical issues and issued specific guidance on DBD and DCD (52, 53). The efforts of the UKDEC are believed to have contributed to the increased rates of DCD in the UK (54). The UKDEC has also issued guidance focused on particularly sensitive areas including infant and paediatric donation (55, 56) and interventions introduced prior to death designed to optimize organ quality (57). Due to widespread budget cuts and the need for increased fiscal austerity, funding for this valuable resource was withdrawn in 2016.

## Discussion

The UK organ donation and transplant program has undergone significant improvement in the past 10 years. Although much remains to be done, the UK offers an example of a complete system with sound governing structures, clear leadership, and well-established professional roles. Centralization and coordination of activities by the NHSBT has proven to be a very effective strategy that is aligned with ongoing and consistent efforts to ensure integration at all levels and nurture a collegial environment in which everyone understands that we are working toward a shared goal. Training and ongoing professional development are core components of the program. Likewise, high standards for data collection and regular reporting are necessary to inform continuous quality improvement. All staff members are encouraged to participate in research projects and audit activities which are clearly understood to be essential tools for guiding policy and future developments.

To the best of our knowledge, this is the first comprehensive case study of the UK organ donation and transplantation program to be published in the academic literature. This case study was informed by the comprehensive conceptual framework devised by Johnston-Webber et al. (9) and drew on a wide range of information sources. Our initial findings were verified and developed further by members of an expert panel, including one individual with specific UK expertise. This effort resulted in a detailed real-life description of the UK program together with an insight into its growth and development over the past two decades. This study highlights features of particular importance to recent improvements in the performance of the UK transplant system.

We acknowledge the limitations of our methodology. The narrative review may have been influenced by the subjective views of the authors. Similarly, the views and insights provided by the expert panel may not be representative of those all professionals working in the field. Likewise, the contexts in which this system developed are in some senses unique to the UK; the lessons learned and insights gained may not be relevant or transferrable to other settings. Additional comparative research on the transplant systems of other countries might help to strengthen the overall impact and transferability of the factors identified in this study (1–3,58, 59).

The creation of a successful organ donation and transplantation program is a highly complex enterprise that involves many different aspects of public policy and requires the support and trust of the general public. Therefore, it is important to perform systematic analyses of these experiences and to identify both positive and negative features of worldwide programs that have been established or remain under development. Many cultural and contextual factors differ profoundly from one setting to another, and policy approaches that were effective in one situation may not be directly transferrable to another. However, we believe that a consideration of the key findings from our review of the UK program may provide valuable insights to other countries and that the elements and principles underlying the successful UK program might apply to other settings if suitably adapted for local preferences and circumstances.

## Data Availability

The original contributions presented in the study are included in the article/supplementary material, further inquiries can be directed to the corresponding author.
